# Treatment patterns in major depressive disorder after an inadequate response to first-line antidepressant treatment

**DOI:** 10.1186/1471-244X-12-143

**Published:** 2012-09-18

**Authors:** Mauro Garcia-Toro, Esteban Medina, Jaime L Galan, Miguel A Gonzalez, Jorge Maurino

**Affiliations:** 1 Department of Psychiatry, Hospital Son Llatzer, Palma de Mallorca, Spain; 2 AstraZeneca Medical Department, Madrid, Spain; 3 Hospital Quirón, Málaga, Spain; 4 Quintiles, Madrid, Spain

**Keywords:** Major depressive disorder, Response, Antidepressant treatment, Augmentation, Switching, Combination

## Abstract

**Background:**

The aim of the study was to determine the most common pharmacological strategies used in the management of major depressive disorder (MDD) after an inadequate response to first-line antidepressant treatment in clinical practice.

**Methods:**

Multicenter, non-interventional study in adult outpatients with a DSM-IV-TR diagnosis of MDD and inadequate response to first-line antidepressant medication. Multiple logistic regression analyses were performed to identify independent factors associated with the adoption of a specific second-line strategy.

**Results:**

A total of 273 patients were analyzed (mean age: 46.8 years, 67.8% female). Baseline mean Montgomery-Asberg Depression Rating Scale total score was 32.1 (95%CI 31.2-32.9). The most common strategies were: switching antidepressant medication (39.6%), augmentation (18.8%), and combination therapy (17.9%). Atypical antipsychotic drugs were the most commonly used agent for augmenting antidepressant effect. The presence of psychotic symptoms and the number of previous major depressive episodes were associated with the adoption of augmenting strategy (OR = 3.2 and 1.2, respectively).

**Conclusion:**

The switch to another antidepressant agent was the most common second-line therapeutic approach. Psychiatrists chose augmentation based on a worse patients’ clinical profile (number of previous episodes and presence of psychotic symptoms).

## Background

Major depression is one of the most prevalent mental disorders in Europe, with an estimated prevalence of 6.9% [1]. According to some projections, major depressive disorder (MDD) will be one of the three leading causes of burden of disease in 2030 [2].

Despite the introduction of newer-generation antidepressants, approximately 50% of patients experience non-response to treatment with a first-line antidepressant [3,4]. The presence of residual symptoms has been associated with a higher risk of recurrence, more chronic depressive episodes, a shorter duration between episodes, and a worse functioning [5-7]. In addition, not achieving symptomatic remission increases the economic burden of this mental disorder which is mainly explained by loss of productivity due to disability and absenteeism and to the use of healthcare resources such as medical visits and pharmacological therapies [8-11].

Current guidelines recommend four pharmacological strategies for the management of partial response or non-response of MDD: increasing the dose of the antidepressant, switching to a different antidepressant, augmenting the treatment regimen with a non-antidepressant agent such as lithium, atypical antipsychotic drugs or thyroid hormones, or combining the initial antidepressant with a second antidepressant [12]. However, the evidence to give clear recommendations regarding the optimal stepwise pharmacological approach after inadequate response to first-line antidepressant treatment remains limited [4,13-15]. Several factors should be considered when choosing between strategies, including the potential loss of partial benefit from the first-line AD and/or the risk of withdrawal symptoms when switching agents, the risk of drug interactions, tolerability and adherence issues with combination and augmentation strategies [4].

In part due to the lack of clear recommendations, there is considerable heterogeneity in real clinical practice in terms of how and when to adopt a new pharmacological approach in those patients who have not responded adequately to first-line treatment. Moreover, little is known regarding factors influencing physician choice among the four proposed strategies.

The primary aim of the study was to determine the most common pharmacological strategies for MDD patients after initial antidepressant treatment failure in clinical practice. In addition, we explored the relationship between sociodemographic and clinical characteristics and the likelihood of a switching, augmentation or combination strategy being adopted by the psychiatrist.

## Methods

This was a 6 months, non-interventional study conducted in 60 community mental health centres throughout Spain. The study was approved by the institutional review board of the Fundació Catalana d´Hospitals (Barcelona, Spain; NCT00782964).

Male or female patients aged 18 or above with a *Diagnostic and Statistical Manual of Mental Disorders, Fourth Edition, Text Revision* (DSM-IV-TR) diagnosis of major depressive disorder (MDD), single or recurrent episode, were eligible for inclusion. Participants were outpatients meeting the following criteria: a history of inadequate response (partial or non-response) during the current episode to first-line treatment with a selective serotonin reuptake inhibitor (SSRI) or a selective serotonin-norepinephrine reuptake inhibitor (SNRI), which was given for at least 6 weeks at adequate doses according to the label. Exclusion criteria included: duration of current MDD episode > 12 months or < 4 weeks, and clinically significant medical illness. Written informed consent was obtained once the study had been fully described to the participants.

Sociodemographic and clinical data, as well as the new pharmacological strategy adopted, were collected from medical records and clinical interviews. The study protocol did not pre-specify any particular therapy. Therefore, the new treatment strategy prescribed was based on the physicians’ criteria only.

Psychometric evaluations included the Montgomery-Asberg Depression Rating Scale (MADRS), the Hamilton Anxiety Rating Scale (HARS), and the Clinical Global Impression - Severity of Illness Scale (CGI-S) [16,17].

### Statistical analysis

The analysis presented in this article is focused on the baseline visit. Patients were allocated to three groups for analysis according to the new treatment strategy adopted: switching, augmentation or combination. Descriptive statistics were obtained for all variables: mean, standard deviation (SD) and 95% confidence interval (CI) for continuous variables, and frequencies and percentages for categorical variables.

A multinomial logistic regression was used to explore the relationship between sociodemographic and clinical characteristics of the patients and the likelihood of adopting a specific therapeutic strategy. First, demographic and clinical characteristics were tested using univariate multinomial logistic regression. Candidate variables with a p-value ≤ 0.2 in the univariate analyses were included in a backward multivariate multinomial logistic regression procedure to evaluate which were independently associated with the treatment strategy adopted by the psychiatrist after first-line failure. Data analysis was performed using SAS V.8.02 (SAS, Cary, NC, USA).

## Results

A total of 336 patients were included in the study. The most commonly used strategy was switching antidepressant medication (N = 133, 39.6%). Meanwhile, 80 (23.8%) patients were allocated to a combination therapy, 60 (17.9%) to an augmentation strategy and 63 (18.8%) to mixed strategies.

The present analysis was conducted on those patients that started a switching, a combination or an augmentation strategy (N = 273). The mean age of this sample was 46.8 years (SD 10.7), and 67.8% were female (Table 1). The mean MADRS total score was 32.1 (95%CI 31.2-32.9). The most common AD medications at baseline were: venlafaxine (15.2%), escitalopram (10.9%), and paroxetine (9.8%). The mean duration of baseline AD treatment was 12 weeks (95%CI 10.6-13.3). A change to an SNRI or an SSRI were the most frequent switching options (63.9% and 25%, respectively). Most typical combinations consisted of an SSRI plus an SNRI (50%), mirtazapine (19.3%), or bupropion (11.4%). Atypical antipsychotic drugs were the most commonly used agents for augmenting antidepressant effect (N = 46/60, 76.6%). The clinical characteristics of the whole sample and of each study group are summarized in Table 2.

**Table 1:** Sociodemographic characteristics of the sample

	**Switching**	**Combination**	**Augmentation**	**Total**
	**(N = 133)**	**(N = 80)**	**(N = 60)**	**(N = 273)**
Age (years), mean (95%CI)	46.5 (44.5-48.4)	45.9 (43.4-48.1)	49.0 (46.5-51.4)	46.8 (45.5-48.1)
Sex (female), n (%)	92 (69.2)	53 (66.3)	40 (66.7)	185 (67.8)
Marital status, n (%)				
Single	16 (12.0)	11 (13.8)	14 (23.3)	41 (15.0)
Married	80 (60.2)	48 (60.0)	35 (58.3)	163 (59.7)
Divorced or separated	37 (27.8)	21 (26.3)	11 (18.3)	69 (25.3)
Education level, n (%)				
No education/ Primary education	78 (58.7)	46 (57.5)	41 (68.3)	165 (60.4)
Secondary/ University education	55 (41.4)	34 (42.5)	19 (31.7)	108 (39.6)
Family structure, n (%)				
Living alone	17 (12.8)	8 (10.0)	11 (18.3)	36 (13.2)
Couple family	17 (12.8)	18 (22.5)	8 (13.3)	43 (15.8)
Parental family	13 (9.8)	9 (11.3)	11 (18.3)	33 (12.1)
Own family	86 (64.7)	45 (56.3)	30 (50.0)	161 (59.0)
Socioeconomic status, n (%)				
Low/Low-Middle	55 (41.4)	30 (37.5)	23 (38.3)	108 (39.6)
Middle	62 (46.6)	40 (50.0)	31 (51.7)	133 (48.7)
Middle-High	16 (12.0)	10 (12.5)	6 (10.0)	32 (11.7)
Work status, n (%)				
Active	79 (59.4)	50 (62.5)	20 (33.3)	149 (54.6)
Unemployed	39 (29.3)	19 (23.8)	20 (33.3)	78 (28.6)
Retired/Pensioner	15 (11.3)	11 (13.8)	20 (33.3)	46 (16.9)

**Table 2:** Clinical characteristics of the sample

	**Switching**	**Combination**	**Augmentation**	**Total**
	**(N = 133)**	**(N = 80)**	**(N = 60)**	**(N = 273)**
Number of previous depressive episodes, mean (95%CI)	1.8 (1.5-2.1)	2.0 (1.5-2.4)	3.5 (2.5-4.4)	2.2 (1.9-2.5)
Previous hospitalization, n (%)	12 (9.0)	10 (12.5)	15 (25.0)	37 (13.6)
Previous suicide attempt, n (%)	20 (15.2)	17 (21.3)	16 (26.7)	53 (19.5)
Family history of psychiatric disorders, n (%)	56 (42.1)	44 (55.0)	30 (50.0)	130 (47.6)
Duration of AD treatment (weeks), mean (95%CI)^*^	12.7 (10.7-14.6)	10.6 (9.1-12.1)	12.2 (8.3-16)	12.0 (10.6-13.3)
Presence of psychotic symptoms, n (%)	3 (2.3)	5 (6.3)	11 (18.3)	19 (7.0)
MADRS total score, mean (95%CI)	31.5 (30.3-32.6)	32.5 (30.9-34.1)	32.8 (30.9-34.6)	32.1 (31.2-32.9)
MADRS Item 10 (suicidality) score, mean (95%CI)	2.0 (1.7-2.2)	2.3 (2.0-2.6)	2.5 (2.2-2.7)	2.2 (2.0-2.3)
HARS total score, mean (95%CI)	24.3 (23.1-25.6)	24.0 (22.3-25.6)	23.0 (21.5-24.5)	24.0 (23.1-24.7)
CGI-S score, mean (95%CI)	4.5 (4.3-4.6)	4.7 (4.5-4.8)	4.7 (4.5-4.9)	4.6 (4.5-4.6)

*Antidepressant treatment for the current MDD episode at enrolment.

MADRS, Montgomery-Asberg Depression Rating Scale; HARS, Hamilton Anxiety Rating Scale; CGI-S, Clinical Global Impression – Severity scale.

Patients allocated to an augmentation strategy presented a worse clinical profile than those allocated to switching or combination therapies in terms of proportion of patients with psychotic symptoms, number of previous depressive episodes and hospitalizations, and MADRS item 10 score (Table 2).

Sociodemographic and clinical variables with a p-value ≤ 0.2 in the univariate analyses were included in the multivariate multinomial logistic regression based on their likely influence on treatment decisions. Only two variables were statistically significant in the multivariate analysis: the presence of psychotic symptoms and the number of previous major depressive episodes (Table 3). Patients with psychotic symptoms and a higher number of previous major depressive episodes were more likely to receive an augmentation strategy (using combination strategy as reference group). Thus, the odds ratio (OR) to receive an augmentation strategy was 3.25 (95%CI: 1.02-10.31) in patients with psychotic symptoms and 1.23 (95%CI: 1.07-1.43) for each previous major depressive episode.

**Table 3:** Independent factors associated with treatment patterns after an inadequate antidepressant response

	**Odds ratio**	**95% CI**	p^*^
Presence of psychotic symptoms			
Switch	0.34	0.08 to 1.48	0.152
Augmentation	3.25	1.02 to 10.31	0.045
Number of major depressive episodes			
Switch	0.96	0.83 to 1.11	0.558
Augmentation	1.23	1.07 to 1.43	0.005

*Results of multivariate multinomial logistic regression. Combination strategy was used as reference group in the analysis.

## Discussion

According to prior studies, the choice of initial antidepressant medication may be influenced by patient characteristics such as older age, illness severity, prior suicide attempts, and presence of a comorbid anxiety disorder [18]. The question of how to proceed with the next step after initially unsuccessful AD treatment is crucial. In our study, switching to a different antidepressant drug was the most common strategy used to treat MDD patients with an inadequate response to first-line treatment (39.6%).

The clinicians’ preference for switching to a different antidepressant has previously been described [19]. Currently, there is not enough clinical evidence to make a clear recommendation about the best next-step treatment in non-responders. However, changing to another class of antidepressant is a favoured option, although the change to another SSRI or to a tricyclic agent could also be used [19,20]. The lower number of drugs involved with switching strategies could avoid potential interactions and assure treatment adherence [12]. Such advantages of switching over the two other strategies may explain its preference among psychiatrists.

Despite the lack of clear evidence-based recommendations, we observed that some clinical characteristics seem to have been considered by psychiatrists when they decided to adopt a new pharmacological approach. Thus, an augmentation strategy seemed to be the preferential therapeutic option for patients with a worse clinical profile. According to the multivariate analysis in our study, patients with more previous depressive episodes and/or psychotic symptoms were more likely to receive augmentation therapy. No randomized controlled trials have directly compared switching and augmentation strategies. The most valuable evidence comes from the Sequenced Treatment Alternatives to Relieve Depression (STAR*D) trial, a four-level study that evaluated different strategies in non-responders [21]. The results of a retrospective analysis comparing switching and augmentation strategies in patients participating in the STAR*D trial has recently been published [22]. Such analysis showed that patients who complete initial treatment of 12 weeks or more and have a partial response with residual mild depressive symptoms may benefit more from augmentation than switching.

The STAR*D trial also showed that patients with longer depressive episodes were less likely to achieve remission [21]. Guidelines drawn up by scientific associations on the management of MDD patients recommend that, when remission is not achieved with an AD after 6 to 8 weeks of treatment at an adequate dose, this should be changed [5,20,23]. The timing of adopting a new pharmacological approach in our study was greater than recommended: mean duration of AD treatment at baseline was 12.0 weeks (SD 11.1). The variability observed by psychiatrists in their clinical attitude towards patients with major depression has previously been described at primary care level [24,25]. A recent publication by Chang et al. found little active management among MDD patients treated in primary care centres in the USA [26]. General practitioners were not more likely to adjust therapy, even when feedback regarding their patients´ symptoms indicated an inadequate response [26].

The fact that approximately one half of patients do not respond to first-line treatment with antidepressants constitutes a major concern for psychiatrists in daily clinical practice [12,27].

Although widely used and usually safe, the efficacy of even the most widely prescribed combinations of antidepressants has not been established by properly controlled clinical trials [14]. Among patients who did not achieve remission with initial citalopram treatment in the STAR*D trial, approximately one-third of patients augmenting treatment and one-quarter of patients switching antidepressant achieved subsequent remission [3].

Lithium is one of the oldest agents used to improve clinical outcomes in patients with inadequate responses from antidepressant trials [12]. However, in our study only 5 patients received lithium (8.3% among the total of augmentation strategies). The adverse event profile, low serum therapeutic index, and long-term risks of thyroid and renal compromise associated with this drug might explain its poor uptake in our sample [4]. Among the different augmentation strategies, augmentation with atypical antipsychotics has been the most studied approach over recent years. The use of adjunctive atypical antipsychotic drugs is the strategy with the largest evidence for AD non-responders with a pooled remission rate of approximately 47% [13]. However, the potential benefits of these medications must be weighed up against their known acute and long-term risks, such as extrapyramidal symptoms and metabolic side effects [4]. In refractory MDD studies, quetiapine extended release 150 mg/d and 300 mg/d had significantly higher risks for sedation: the numbers needed to treat to harm (NNTH) were 9 and 7, respectively [28]. Aripiprazole adjunctive therapy to antidepressants resulted in a significantly higher risk for akathisia relative to placebo (NNTH of 5, 95%CI 4–7) [28]. In addition, fluoxetine plus olanzapine resulted in significantly greater weight gain and increase in total cholesterol compared with fluoxetine monotherapy [4].

The present manuscript has several limitations. First, our study analyzed a total of 273 patients and probably this small sample size could have limited the finding of additional associations. Second, we only analyzed outpatients receiving care from psychiatrists. Therefore, our findings may neither generalize to mild depressive patients, mainly managed in a primary care setting, nor the inpatient population. Third, comorbidity, ethnicity, and adverse events associated with the first antidepressant were not assessed, which may also be relevant factors affecting MDD management.

## Conclusions

In our study, the switch to another antidepressant was the most common therapeutic approach (39.6%) performed by psychiatrists. The number of previous episodes and the presence of psychotic symptoms were associated with the adoption of augmenting with a non-antidepressant agent.

However, limited evidence is available to guide clinical decision making after initial treatment failure. The identification of factors related to the efficacy of a specific treatment would offer clinicians the opportunity to more adequately select patients who are eligible for such strategy. Further studies are needed to help determine evidence-based options for the management of MDD patients after an inadequate response to first-line antidepressant treatment.

## Competing interests

This study was sponsored by AstraZeneca Spain. EM and JM are employees of AstraZeneca Spain. JLG was an employee of AstraZeneca Spain when the study was designed and the data were collected. MAG is an employee of Quintiles Spain. MG-T has no conflict of interest to declare.

## Authors´ contributions

MG-T and JLG conceived the study design. EM and MAG performed the statistical analysis. All authors made meaningful contributions to data inter pretation. MG-T, EM, and JM cowrote the final draft of the manuscript. All authors read and approved the final manuscript.

## Pre-publication history

The pre-publication history for this paper can be accessed here:

http://www.biomedcentral.com/1471-244X/12/143/prepub
